# Use of Complementary and Alternative Medicine in the General Public of Western Saudi Arabia: A Cross-Sectional Survey

**DOI:** 10.7759/cureus.32784

**Published:** 2022-12-21

**Authors:** Syed Faisal Zaidi, Abdullah Alzahrani, Zackary Alghamdy, Dheyya Alnajar, Nawaf Alsubhi, Aslam Khan, Mohamed E Ahmed

**Affiliations:** 1 Faculty of Eastern Medicine, Hamdard University Islamabad Campus, Islamabad, PAK; 2 College of Medicine, King Saud Bin Abdulaziz University for Health Sciences College of Medicine, Jeddah, SAU; 3 Riphah Institute of Pharmaceutical Sciences, Riphah International University, Lahore, PAK; 4 Biostatistics, King Abdullah International Medical Research Center, Jeddah, SAU

**Keywords:** jeddah, saudi arabia, self-use, awareness, barriers, education level, alternative medicine, complementary medicine

## Abstract

Background: Complementary and alternative medicine (CAM) has been continuously used worldwide. Various cultures have used this path of healing, and to our date, people are still using it and some even prefer it to modern medicine. Thus, this study aims to analyze awareness, self-use, perceptions, beliefs, and attitudes toward CAM in the general public of Jeddah, Saudi Arabia.

Methods: A descriptive observational cross-sectional study was conducted in the public places of Jeddah. Data were collected from 784 participants using a self-administered paper-based questionnaire, and statistical analysis was performed using Statistical Package for the Social Sciences (SPSS).

Results: The majority of the population was aware of massage (91.8%), herbs (90.7%), nutritional supplements (89.8%), and prayers (88.1%). With regards to usage and effectiveness, prayers and spirituality is used by 75.5% of the population and considered to be the most effective by 76.0%. Respondents obtained information about CAM mostly from friends and relatives (76.6%), followed by media (67.2%), while lack of knowledge about CAM and lack of trained professionals are the most perceived barriers to CAM implementation. Data showed a significant association (p < 0.05) between gender, awareness, and self-use of CAM modalities. Yoga (44.2%) and herbs (72.6%) were mostly used by females, whereas males were mostly aware of cupping (90.4%) and cauterization (76.2%). Another significant association was found between the level of education, awareness, and self-use of CAM modalities indicating that those who were not educated were aware of and used cauterization the most, while those who went to college were more aware of yoga (75.4%). Lastly, having a relative in the healthcare field showed a significant association with awareness of yoga, prayers, and spirituality compared to other CAM modalities.

Conclusion: In conclusion, the present study revealed that the majority of the Western Saudi Arabian population was aware of several CAM modalities and practiced some form of CAM. However, awareness of specific types of CAM may relate to gender, educational level, and relationship to the medical field.

## Introduction

Complementary and alternative medicine (CAM) is the use of traditional or non-conventional medicine to treat certain diseases, especially chronic diseases. CAM includes practices that go along with standard medical care or substitute it completely. “Complementary” medicine can include combining a form of traditional medicine with a conventional one, for example, the use of acupuncture to help lessen some side effects of conventional medical treatment. Alternative medicine entails replacing standard medical care completely with a technique or approach not considered part of conventional medicine [1].

The use of CAM has been continuously growing worldwide among adults and children. In the United States, studies showed that the use of yoga, meditation, and chiropractors had increased approximately from 22% in 2012 to 38% in 2017 among adults, and from 7% to 17% among children during that period [2,3]. One of the hallmarks of CAM is the use of herbs for the treatment and prevention of several diseases. 87% of the counted global population’s diseases are treated by drugs that are plant-based [4]. There is an exponential growth in the interest in CAM, particularly in herbal medicine and treatment [5].

Many countries around the world practice CAM, especially Islamic countries, including the Kingdom of Saudi Arabia (KSA). CAM in Islamic countries is derived from either religious belief (Holy Quran and Hadith), such as honey, black seeds, cauterization, and hijama, or cultural/traditional beliefs, such as coconut oil and massage. The safety of these CAM modalities and how they might delay patients seeking treatment has not been clarified. So, the Saudi government has established an act to govern the practice of CAM and encourage evidence-based CAM, “An act was issued by Royal Decree M/18 dated 18/3/1398 H (equivalent to 26 February 1978). Articles 44 and 50 of this act, prohibit the handling of locally produced or imported products before their registration with the Ministry of Health” [6]. However, only a few studies have been conducted on CAM in KSA to examine its extensive usage among the general public.

Many countries have now included several plant-based treatments in their pharmacopeias to help with the management of several diseases like migraine, headaches, and many others [7,8]. With this increased interest in CAM and its potential benefit, it is important to make sure that the general public takes advantage of the research-based, safe, and certified use of CAM, and does not expose themselves to the negative risks. It has been shown that those with strong beliefs about CAM use were likely to have a positive attitude toward using it. It was also shown that many patients do not disclose CAM use to their doctors, which can influence the treatment and management of the patient [9].

By learning the attitude of the population, we can have a better understanding of their likelihood of CAM use. Thus, in this study, a questionnaire was used to measure general public perception, knowledge, understanding, and use of CAM and describe the factors that are associated with their attitude and possible reasons to use CAM.

## Materials and methods

Ethical approval and consent to participate

This study was accepted by the Research Committee and approved by Institutional Review Board (IRB), King Abdullah Medical Research Center (KAIMRC), Ministry of National Guard-Health Affairs (MNG-HA), with study number SP19/202/J and Memo. Ref. no. IRBC/0770/19.

Study design, area, and settings

This is a cross-sectional study, where a multiple-choice questionnaire was issued to target the general public of Jeddah in Western Saudi Arabia. Individuals aged 18 years and above were eligible and both genders were recruited. The questionnaire consisted of six parts: the aim of the first part was designed to assess the general demographic data, the second part was to analyze awareness and use of CAM, the third part was to evaluate the perception of CAM, the fourth part was to assess sources of information, the fifth part was to evaluate perceived barriers to CAM and the sixth section was to appraise beliefs and attitudes towards CAM.

Cochran’s equation of simple random sampling which depends on population proportion was used to determine the sample size, the equation takes the form:

$n* =(z^2 x p (1-p))/d^2$

by taking p = 50%, which gives the maximum possible sample size, and d = 0.05 then, the initial sample size will be:

$n* =(2^2 x 0.5 (1-0.5))/〖0.05〗^2 =400$

However, in the actual field survey, clustered random sampling was used, this required multiplying the sample size by the design effect which was taken to be two. Therefore, the actual sample size was 800. The actual sample size was distributed among selected subareas. Cluster random sampling was used by dividing the study population into subareas, randomly selecting some of the subareas, and dividing the sample size proportionately among the selected subareas.

Data collection process

A self-administered paper-based questionnaire was used in this survey. We validated the questionnaire by determining face validity from experts in biostatistics and health care. The content validity was done by the subject expert. Eventually, a pilot study (30 participants) was performed to check for the clarity of the questions, and modifications were made to the questionnaire complying with the feedback from the pilot testing. Arabic translation has been checked by a language expert.

The researchers collected the data at multiple malls, parks, stores, etc. giving a questionnaire to a sample of the general public. The questionnaire was distributed to the general public who gave consent for filling the questionnaire. 10 minutes were given to fill out the questionnaire and the researcher was providing help and explanation if needed. After the sample number was achieved, the data were saved on a personal computer with a password while respondents' identities were kept anonymous as well to insure confidentiality.

Data analysis

After data collection, the data were entered into Microsoft Excel. Then, the data was transferred to Statistical Package for the Social Sciences (SPSS) software version 22.0 for data analysis. Qualitative variables, like gender, were presented by frequency, percentage, and bar graph. Quantitative variables were presented as mean and standard deviation. Independent t-test, chi-square test, and analysis of variance were used for data comparison. The dependent variable in this study was CAM perception and use, and the independent variables were demographics, source of information, level of education, and beliefs about CAM. P-value <0.05 was taken as significant.

## Results

The questionnaires were distributed in different malls and parks in the city of Jeddah. A total of 1,000 surveys attached to informed consent were printed and ready to be distributed. Seven hundred and eighty-four were fully completed. Forty-nine had either an unsigned informed consent or an unfilled survey and thus could not be used for this study. There were about 167 people who rejected being involved in this study. This has made the response rate equal to 78.4%.

The basic information of the participants is shown in Table 1. Male participants make up 49.2% of the total sample size, while females make up 50.8%. Regarding education, 63.9% of the sample had secondary education, 18.1% had middle school, 16.2% had a college education, 0.9% had primary education and the same percentage was non-educated. 85.6% of the participants were Saudis, while the rest held different nationalities. Also, 48.9% of the participants had relatives that work in the healthcare field. Moreover, the average age of the participants was 31.07 with a standard deviation of 10.8.

**Table 1:** Basic demographic information of the population

Variable		N=784
Gender	Male	386 (49.2%)
	Female	398 (50.8%)
Education	None	7 (0.9%)
	Primary	7 (0.9%)
	Middle	152 (18.1%)
	Secondary	501 (63.9%)
	College/University	127(16.2%)
Age	Mean:	31.1
	Standard Deviation:	10.8
Nationality	Saudi	671 (85.6%)
	Non-Saudi	113 (14.4%)
Family related to health care	Yes	383 (48.9%)
	No	401 (51.1%)

Table 2 shows that most of the population is aware of massage, herbs, Nutritional supplements, and Prayers. Aromatherapy, on the other hand, was the least to be recognized by the participants. Regarding self-use, 75.5% of the population have used prayers and spirituality making it the top CAM modality used by the study population. Aromatherapy was the least to be used followed by Acupuncture.

**Table 2:** The pattern of awareness, self-use, and perceived effectiveness and harmfulness of different CAM modalities CAM: Complementary and alternative medicine

CAM modality	Awareness N(%)	Self-use N(%)	Effectiveness (0-5)(%)	Harmfulness (0-5)(%)
Acupuncture	563 (71.8%)	164 (20.9%)	1.88 (38%)	1.47 (30%)
Yoga	558 (71.2)	271 (34.5%)	2.13 (42%)	1.14 (22%)
Cupping	690 (88%)	336 (42.8%)	2.92 (58%)	1.43 (28%)
Aromatherapy	303 (39.6%)	144 (18.3%)	1.42 (28%)	0.93 (18%)
Cauterization	592 (75.5%)	256 (32.7%)	2.47 (49.4%)	1.80 (36%)
Herbs	711 (90.7%)	514 (65.6%)	3.42 (68%)	1.63 (32%)
Massage	720 (91.8%)	560 (71.5%)	3.59 (72%)	1.33 (26%)
Nutritional supplements	704 (89.8%)	525 (67%)	3.33 (66%)	1.60 (30%)
Prayers and spiritualties	691 (88.1%)	592 (75.5%)	3.84 (76%)	0.90 (18%)

The Likert scale (1-5) was used to assess the effectiveness and harmfulness of CAM modalities. Of all CAM practices, prayers and spirituality (76%) were thought of as the most effective practice followed by massage (72%). However, aromatherapy (28%) and acupuncture (38%) were perceived to be the least effective modalities. Regarding harmfulness, cauterization (36%) was perceived to be the most harmful practice followed by the use of herbs (32%). Aromatherapy (18%) and prayers and spiritualties (18%), nevertheless, were perceived to be the least harmful according to the participants.

According to Table 3, most of the participants considered friends and relatives as their preferred sources when using CAM. The media was the second most reliableLikerte regarding the use of CAM. The participants considered formal CAM courses or training as the least favorite source to use when practicing CAM.

**Table 3:** Sources of CAM information CAM: Complementary and alternative medicine

Source	YES N (%)
Books	464 (59.2%)
Media	527 (67.2%)
CAM practitioners	416 (53.2%)
Formal CAM course or training	258 (32.9%)
Other health professionals	475 (60.7%)
Friends and relatives	601 (76.7%)

Table 4 illustrates the participants' perceptions of the barriers to CAM implementation. The majority of people believe that a lack of knowledge about CAM is hindering CAM's success. The second obstacle was the lack of trained professionals.

 

**Table 4:** Perceived barriers to CAM implementation CAM: Complementary and alternative medicine

Statement	Scale mean (% agreement)
Lack of trained professionals	4.0 (80)
Lack of scientific evidence	3.7 (74)
Long time for treatment	3.5 (70)
Lack of knowledge about CAM	4.1 (82)

Table 5 demonstrates the Likert scale mean of the respondents regarding their beliefs and attitudes toward CAM. Participants tended to agree that people are more likely to use CAM if their teachers or friends and relatives discuss it with them. They also agreed that the more knowledge a person has about CAM, the more likely he/she is to use it.

**Table 5:** Beliefs and attitudes towards CAM CAM: Complementary and alternative medicine

Statement	Scale mean (%)
CAM providers give good information on maintaining a healthy lifestyle	3.5 (70)
The more knowledge a person has about CAM, the more likely he is to use it	4.0 (80)
People are more likely to use CAM if his/her friends or relatives discuss it with them	4.0 (80)
People are more likely to use CAM if his/her teachers discuss it with them	4.0 (81)
Believing on mental and spiritual aspect of health encourages the use of CAM	3.9 (78)
CAM involves natural plant formula which are healthier than taking drugs given by the medical doctor	3.6 (72)

Table 6 shows the Chi-Square results for the association between gender, awareness, and self-use of CAM modalities. Regarding awareness, a significant association of gender with cupping, aromatherapy and cauterization was noticed. Males tend to be more aware of cupping and cauterization than their counterparts. Females, however, were found to be more aware of aromatherapy than men. As shown in the other part of Table 6, gender seemed to affect the self-use of acupuncture, yoga, cupping, herbs, and nutritional supplements. Females tend to use those modalities more than men except for cupping.

**Table 6:** Association between gender, awareness, and self-use of CAM modalities CAM: Complementary and alternative medicine

Modality	Awareness			Self-use		
	N (%) male	N (%) female	P-value	N (%) male	N (%) female	P-value
Acupuncture	386 (70.3%)	292 (73.4%)	0.210	58 (15.1%)	106 (26.7%)	0.001
Yoga	263 (68.2%)	295 (74.1%)	0.171	95 (24.6%)	176 (44.2%)	0.001
Cupping	349 (90.4%)	341 (85.7%)	0.001	184 (47.7%)	152 (38.2%)	0.023
Aromatherapy	134 (34.7%)	169 (42.5%)	0.001	59 (15.3%)	85 (21.4%)	0.088
Cauterization	294 (76.2%)	298 (74.9%)	0.001	117 (30.3%)	139 (34.9%)	0.341
Herbs	344 (89.1%)	367 (92.2%)	0.119	224 (58.3%)	289 (72.6%)	0.001
Massage	358 (92.7%)	362 (91%)	0.652	273 (70.7%)	287 (72.1%)	0.812
Nutritional supplements	347 (89.9%)	357 (89.7%)	0.459	243 (63%)	282 (70.9%)	0.042
Prayers and spirituality	341 (88.3%)	350 (87.9%)	0.626	284 (73.6%)	308 (77.4%)	0.406

Table 7 demonstrates the relationship between the level of education and awareness and self-use of CAM modalities. There was no association between the level of education and CAM modality awareness except for Yoga, cupping, and cauterization. College students were more aware of Yoga, and cauterization than people with lower education levels. People with secondary education were found to be more aware of cupping than those with lower levels and college education. Self-use, however, was found to have a significant association with the level of education in yoga, cauterization, herbs, and massage. Participants who had a secondary level of education were found to be using yoga the most. Uneducated participants have used cauterization more than educated participants. People who are either uneducated or have a primary level of education tended to use herbs more than their counterparts. The massage modality was mostly used by people with a primary level of education.

**Table 7:** Association between education, awareness, and self-use of CAM CAM: Complementary and alternative medicine

CAM modality	Awareness						Self-use					
	None (%)	Primary (%)	Middle (%)	Secondary (%)	College (%)	P-value	None (%)	Primary (%)	Middle (%)	Secondary (%)	College (%)	P-value
Acupuncture	3 (42.9)	3 (42.9)	100 (70.4)	366 (73)	91 (71.7)	0.183	1 (14.3)	0 (0)	28 (19.7)	103 (20.6)	32 (25.2)	0.142
Yoga	3 (42.9)	0 (0)	99 (68.8)	378 (69.7)	78 (75.4)	0.001	2 (28.6)	1 (14.3)	49 (34.5)	196 (39.2)	23 (18.1)	0.001
Cupping	5 (71.4)	4 (57.1)	126 (88.7)	452 (90.3)	103 (81.1)	0.002	2 (28.6)	3 (68.1)	56 (39.4)	214 (42.7)	61 (48.1)	0.054
Aromatherapy	2 (28.6)	2 (28.6)	54 (38)	187 (37.4)	58 (45.7)	0.244	0 (0)	3 (42.9)	21 (14.8)	104 (20.8)	16 (12.6)	0.107
Cauterization	7 (100)	4 (57.2)	107 (75.4)	368 (73.4)	106 (83.5)	0.036	5 (71.4)	3 (42.9)	58 (40.8)	149 (29.8)	41 (32.3)	0.002
Herbs	7 (100)	7 (100)	127 (89.4)	454 (90.6)	116 (91.3)	0.085	6 (85.8)	6 (85.8)	104 (73.3)	330 (64.1)	68 (53.6)	0.001
Massage	5 (71.5)	6 (85.7)	128 (90.1)	466 (93)	115 (90.5)	0.175	3 (57.2)	6 (85.7)	104 (73.2)	367 (73.3)	79 (62.2)	0.002
Nutritional supplements	7 (100)	5 (71.4)	124 (87.3)	451 (90)	117 (72.1)	0.529	5 (71.4)	4 (57.2)	91 (64.1)	344 (68.6)	81 (63.7)	0.005
Prayers and spirituality	6 (85.7)	7 (100)	124 (87.4)	437 (87.3)	117 (92.1)	0.426	5 (71.4)	7 (100)	109 (76.8)	383 (76.5)	88 (69.3)	0.376

According to Table 8, which includes the results of the Chi-square test, there was no association found between having a relative in the healthcare field and CAM modalities awareness except for yoga, prayers, and spirituality. Those who do have a relative in the healthcare field were less aware of yoga, prayers, and spirituality as CAM practices. There was no relationship at all between having a relative in the healthcare field and the self-use of CAM modalities.

**Table 8:** Association of having a relative in the healthcare field with awareness and the self-use of CAM CAM: Complementary and alternative medicine

CAM modality	Awareness (%)			Self-use (%)		
	Yes	No	P-value	Yes	No	P-value
Acupuncture	280 (73.1)	283 (70.6)	0.526	85 (22.2)	79 (19.7)	0.523
Yoga	284 (74.2)	274 (68.3)	0.028	131 (34.2)	140 (34.9)	0.855
Cupping	341 (89)	349 (87.1)	0.533	162 (42.3)	174 (43.4)	0.769
Aromatherapy	151 (39.4)	152 (37.9)	0.869	68 (17.7)	76 (18.9)	0.784
Cauterization	300 (75.7)	302 (75.3)	0.973	122 (31.8)	134 (33.4)	0.067
Herbs	346 (90.3)	365 (91)	0.946	253 (66.1)	261 (65.1)	0.923
Massage	351 (91.6)	369 (92)	0.977	279 (72.8)	281 (70.1)	0.221
Nutritional supplements	354 (89.8)	360 (89.7)	0.167	260 (67.9)	265 (66.1)	0.855
Prayers and spirituality	328 (85.7)	354 (90.5)	0.003	286 (74.7)	306 (76.3)	0.818

## Discussion

The results of this study indicate that the majority of the participants were both aware of and have self-used one or more modalities of CAM. Given that Saudi culture is heavily influenced by the religion of Islam [10], it was of no surprise that the data and results from our study indicated that prayer/spirituality was the most used form of CAM and perceived to be the least harmful and most effective. Also, when comparing this to data from similar studies, the same pattern was noticed. For instance, a study done to show the patterns of CAM use in African Americans showed that prayer was the most commonly used form of CAM [11]. Although aromatherapy was considered the least known modality (39.6%), it was also considered one of the least harmful modalities along with prayers and spiritualties, followed by yoga and massage. This could be explained by the fact that those CAM modalities are non-invasive or physically challenging.

In terms of the participants' sources of CAM information, the most common source was friends and relatives, followed by the media, a finding which is strengthened when comparing our results to a study done in Riyadh where about 85% of Saudi patients sought health-related information through sources like WhatsApp, YouTube, and Facebook [12]. Another explanation of this finding is that the heavy impact of family and culture in an Islamic society is widely noticeable. This influence can be seen in other Islamic regions as demonstrated in previous studies from Pakistan and the Majmaah Province of Saudi Arabia [13,14]. In contrast, CAM practitioners and formal CAM courses or training are the least used sources of CAM information for the participants.

Perceived barriers to CAM implementation were mainly due to a lack of trained professionals and a lack of knowledge about CAM. In fact, a study conducted on CAM among Saudis documented that most of the healers providing CAM modalities were illiterate and lack an official degree [15]. The findings of this study showed that the majority of the participants believed that the lack of knowledge about CAM and the lack of trained professionals are hindering CAM success. Therefore, emphasizing the importance of formalizing the field and practice of CAM with trained professionals.

In this study, results demonstrated an association between gender and self-use, with females predominantly participating more in CAM use. The results were especially significant, with a p-value < 0.05, in acupuncture, yoga, nutritional supplements, and the use of herbs as alternative medicine. These findings fall in line with a previous study done on CAM practices that were conducted in Europe, which shows a higher proportion of CAM users were females [16]. However, males expressed more self-use of cupping with a similarly significant p-value.

Regarding the level of education and awareness and self-use of CAM, variable results were found in the literature. It was evident from this study that those with low to no education were more likely to use CAM. On the contrary, a study done in Iceland showed opposite findings concerning education and self-use, the higher the education the more likely they were to participate in CAM use [17]. In this study, participants with no education were found to be more likely to use herbs and cauterization than those with higher education. However, a study conducted in Turkey showed no significant relationship between the level of education and herbal product consumption [18]. Contrary to that, another study done on breast cancer patients showed those with higher levels of education are more likely to use herbal products [19]. These disparities need to be elaborated further with comparative and multi-center studies within the country and across the globe.

Strength

This is the first study done in the general public of Jeddah in Western Saudi Arabia in assessing the awareness, self-use, perceptions, beliefs, and attitudes toward CAM in addition to assessing any association between demographics and awareness or self-use.

Limitations

Since this study was done in a cross-sectional format, some aspects might limit our research including the study design itself, which only provides a snapshot of people's perspectives at a certain time, which could change. Also, despite having a fair sample size and wide coverage of Jeddah’s district, some participants might have been non-residents of Jeddah city and their input on the topic of CAM is different from those residing in Jeddah due to the cultural differences from one city to another. Therefore, in the next study covering our topic, the place of residency needs to be taken into account. In addition, these results are not generalizable to other countries where CAM practices and beliefs may differ.

## Conclusions

The study revealed that a vast majority of Western Saudi Arabia is aware of several CAM modalities and a large proportion of the population practices different types of CAM. In addition, multiple variables were associated with increased self-use of certain CAM modalities and awareness, including education, gender, age, and nationality. This study emphasizes the importance of the demand for more research on the different types of CAM modalities and the dire need of implementing programs to certify professionals and regulate the use of CAM.
